# Regulatory considerations for medical imaging AI/ML devices in the United States: concepts and challenges

**DOI:** 10.1117/1.JMI.10.5.051804

**Published:** 2023-06-23

**Authors:** Nicholas Petrick, Weijie Chen, Jana G. Delfino, Brandon D. Gallas, Yanna Kang, Daniel Krainak, Berkman Sahiner, Ravi K. Samala

**Affiliations:** a U.S. Food and Drug Administration, Center for Devices and Radiological Health, Office of Science and Engineering Labs, Silver Spring, Maryland, United States; b U.S. Food and Drug Administration, Center for Devices and Radiological Health, Office of Product Evaluation and Quality, Silver Spring, Maryland, United States

**Keywords:** AI/ML, regulatory concepts, medical imaging, assessment methods, regulatory science

## Abstract

**Purpose:**

To introduce developers to medical device regulatory processes and data considerations in artificial intelligence and machine learning (AI/ML) device submissions and to discuss ongoing AI/ML-related regulatory challenges and activities.

**Approach:**

AI/ML technologies are being used in an increasing number of medical imaging devices, and the fast evolution of these technologies presents novel regulatory challenges. We provide AI/ML developers with an introduction to U.S. Food and Drug Administration (FDA) regulatory concepts, processes, and fundamental assessments for a wide range of medical imaging AI/ML device types.

**Results:**

The device type for an AI/ML device and appropriate premarket regulatory pathway is based on the level of risk associated with the device and informed by both its technological characteristics and intended use. AI/ML device submissions contain a wide array of information and testing to facilitate the review process with the model description, data, nonclinical testing, and multi-reader multi-case testing being critical aspects of the AI/ML device review process for many AI/ML device submissions. The agency is also involved in AI/ML-related activities that support guidance document development, good machine learning practice development, AI/ML transparency, AI/ML regulatory research, and real-world performance assessment.

**Conclusion:**

FDA’s AI/ML regulatory and scientific efforts support the joint goals of ensuring patients have access to safe and effective AI/ML devices over the entire device lifecycle and stimulating medical AI/ML innovation.

## 1 Introduction

Devices incorporating artificial intelligence and machine learning (AI/ML) can be found in many areas of medicine, and the U.S. Food and Drug Administration (FDA) and Center for Devices and Radiological Health (CDRH) have a history regulating medical AI/ML technologies.^1^ As an example, a semi-automated cervical cytology slide reader incorporating neural network processors was first approved by the FDA in 1995.^2^ FDA receives a high volume of premarket submission inquiries for products leveraging AI/ML technologies, and we expect this trend to continue going forward. Q-submissions are a mechanism for device developers to ask questions and obtain official FDA feedback prior to a formal premarket submission.^3^ Q-submissions are highly recommended to help address a question prior to a full device submission.

The International Medical Device Regulators Forum defined software as a medical device (SaMD)^4^ as “software intended to be used for one or more medical purposes that perform these purposes without being part of a hardware medical device.” While not all SaMD incorporates AI/ML and not all devices involving AI/ML are SaMD, many AL/ML devices are developed and implemented independently from the image acquisition and display devices such that they fit under the wider SaMD umbrella.

Radiology has been a pioneer in adopting AI/ML-enabled devices into the clinical environment. Radiological AI/ML devices are numerous and expanding with applications aiming to improve the efficiency, accuracy, or consistency of the medical image interpretation process across a wide range of radiological tasks and imaging modalities. A non-exhaustive list of AI/ML-enabled medical devices authorized for marketing in the United States and identified through FDA’s publicly available information can be found on the FDA webpage.^5^ Table 1 defines some common types or classes of medical imaging AI/ML that have been submitted to the FDA. Each product type, and its associated product code, contains one or more devices authorized for marketing in the United States with most of these devices considered SaMD.

**Table 1:** A cross section of device product codes that included medical imaging AI/ML. Each product type, and its associated product code, contains at least one AI/ML medical device authorized for marketing in the United States.

FDA product code	Device	Short definition
OMJ^6^	Chest x-ray computer aided detection	Software device to assist radiologists in the review of chest radiographic images and highlight potential nodules that the radiologist should review
QDQ^7^	Radiological computer assisted detection/diagnosis software for lesions suspicious for cancer	An image processing device intended to aid in the detection, localization, and characterization of lesions suspicious for cancer on acquired medical images (e.g., mammography, MR, CT, ultrasound, radiography)
QAS^8^	Radiological computer-assisted triage and notification software	An image processing device intended to aid in prioritization and triage of time-sensitive patient detection and diagnosis based on the analysis of medical images acquired from radiological signal acquisition systems
QJU^9^	Image acquisition and/or optimization guided by artificial intelligence	A device that is intended to aid in the acquisition and/or optimization of images and/or diagnostic signals
QNP^10^	Gastrointestinal lesion software detection system	A computer-assisted detection device used in conjunction with endoscopy for the detection of abnormal lesions in the gastrointestinal tract
QPN^11^	Software algorithm device to assist users in digital pathology	An *in vitro* diagnostic device intended to evaluate acquired scanned pathology whole slide images

AI/ML-enabled medical devices present many opportunities for improving medical practice through their ability to learn from real-world data, improve performance over time, and simplifying the image interpretation process for the clinician by automating routine and tedious tasks. However, these devices come with unique challenges, including the need for large and representative datasets, propagation of biases within the training data and data sourcing process, understanding the upstream and downstream role and impact on clinical workflows, and difficulties assuring continued safety and effectiveness over time for fixed models, e.g., because of changes to the clinical population, and for continuously learning AI/ML devices.

The agency is striving to address challenges around AI/ML development and assessment to ensure patients have access to safe and effective AI/ML devices. In addition to a high-level white paper on good machine learning practices,^12^ the agency is developing regulatory policies,^13^ conducting regulatory science research, and collaborating with stakeholders to better understand and characterize AI/ML models and develop least-burdensome assessment methods.^14^ Similarly, other groups and organizations are working to develop consensus best practices for medical imaging AI/ML.^15^^–^^18^ Some of the more wide-ranging efforts specific to medical imaging AI/ML include the FUTURE-AI guiding principles developed by five European AI in Health Imaging projects^15^ and the American Association of Physicists in Medicine Task Group Report 273 discussing best practices for medical imaging computer-aided diagnosis.^16^

In this review paper, we introduce the reader to the medical device regulatory framework within the United States and provide an overview of common elements included in regulatory submissions that incorporate AI/ML models in medical imaging in Sec. 2.1. Specifically, we discuss the model description, data, nonclinical testing and multi-reader, multi-case studies used to evaluate the device in the hands of the end user in Secs. 2.2–2.5. Finally, we discuss ongoing and planned activities adapting FDA regulatory processes to AI/ML device submissions and how the agency is addressing regulatory science gaps in Sec. 3 of this paper.

## 2 Methods

### 2.1 Regulatory Framework

The FDA regulates medical device manufacturers based on the level of risk posed by the device, which is informed by the intended use of the device, the indications for use of the device, and the technological characteristics of the device. The intended use describes the general purpose of the device or its function while the indications for use are more specific, describing the disease or condition the device will diagnose, treat, prevent, cure, or mitigate, including a description of the patient population for which the device is intended.^19^ The intended use is important in determining both the regulatory pathway and what data and information are necessary in a regulatory submission of that device.

#### 2.1.1 Product classification and regulatory controls

The FDA classifies medical devices into classes I, II, or III, where the class is based on the device risk and determines the extent of regulatory controls necessary to provide a reasonable assurance of the safety and effectiveness for the device (21 C.F.R. § 860). All devices, regardless of class, must adhere to the general control provisions of the Food, Drug, and Cosmetic Act that relate to adulteration; misbranding; device registration as well as device listing; premarket notification, banned devices; notification, including repair, replacement, or refund; records and reports; restricted devices; and good manufacturing practices.^20^ Most medical image processing devices, with or without AI/ML, are currently classified as class II. Aside from some exemptions and in addition to general controls, to market a class II device, a manufacturer must describe and test their device according to all applicable special controls^21^ and demonstrate substantial equivalence between their new device and a legally marketed device, i.e., the predicate device.^19^ Substantial equivalence is established with respect to intended use, design, energy used or delivered, materials, performance, safety, effectiveness, labeling, biocompatibility, standards, and other applicable characteristics in a Class II Premarket Notification [510(k)] submission. Many of these characteristics are described in the product classification of the predicate.^22^ If the device is determined to be substantially equivalent, the manufacturer is then “cleared” to market that device in the United States.

#### 2.1.2 De Novo pathway

The De Novo pathway provides a pathway to a class I or class II classification for medical devices for which general controls or general and special controls provide a reasonable assurance of safety and effectiveness, but for which there is no legally marketed predicate device.^23^ This pathway was created as an alternative to the default high-risk class III classification. Granting of a De Novo establishes a new regulation not only for the specific device but also more broadly for the general device type and intended use, which will then be used for regulation of subsequent devices, e.g., only the first one is a De Novo. Multiple AI/ML device types have been classified as class II through the De Novo pathway, establishing specific, published special controls for the appropriate product class. Example product classes are computer-aided detection (CADe) for lesions using optical colonoscopy^24^ and radiological computer-aided diagnosis (CADx) for lesions suspicious for cancer.^25^ De Novo submissions can also establish broader product classes, as with the first digital pathology AI/ML device, which is more broadly defined as “software algorithms to provide information to the user about presence, location, and characteristics of areas of the image with clinical implications. Information from this device is intended to assist the user in determining a pathology diagnosis.”^26^

#### 2.1.3 Predicates

Choosing a predicate device for a 510(k) submission is an important first step in pursuing marketing clearance for a class II device. As mentioned, the predicate establishes the product classification, special controls, and the safety and effectiveness information necessary to determine substantial equivalence. As such, we recommend manufacturers engage with CDRH reviewers early through the Q-submission program^3^ when identifying a predicate is not clear or when they have other review process questions. The Q-submission process can allow manufacturers to determine if the FDA agrees with their suggested product class or predicate and can be used to address specific questions a manufacturer may have about the testing necessary to demonstrate safety and effectiveness.

#### 2.1.4 AI/ML premarket submissions

**Fig. 1:**
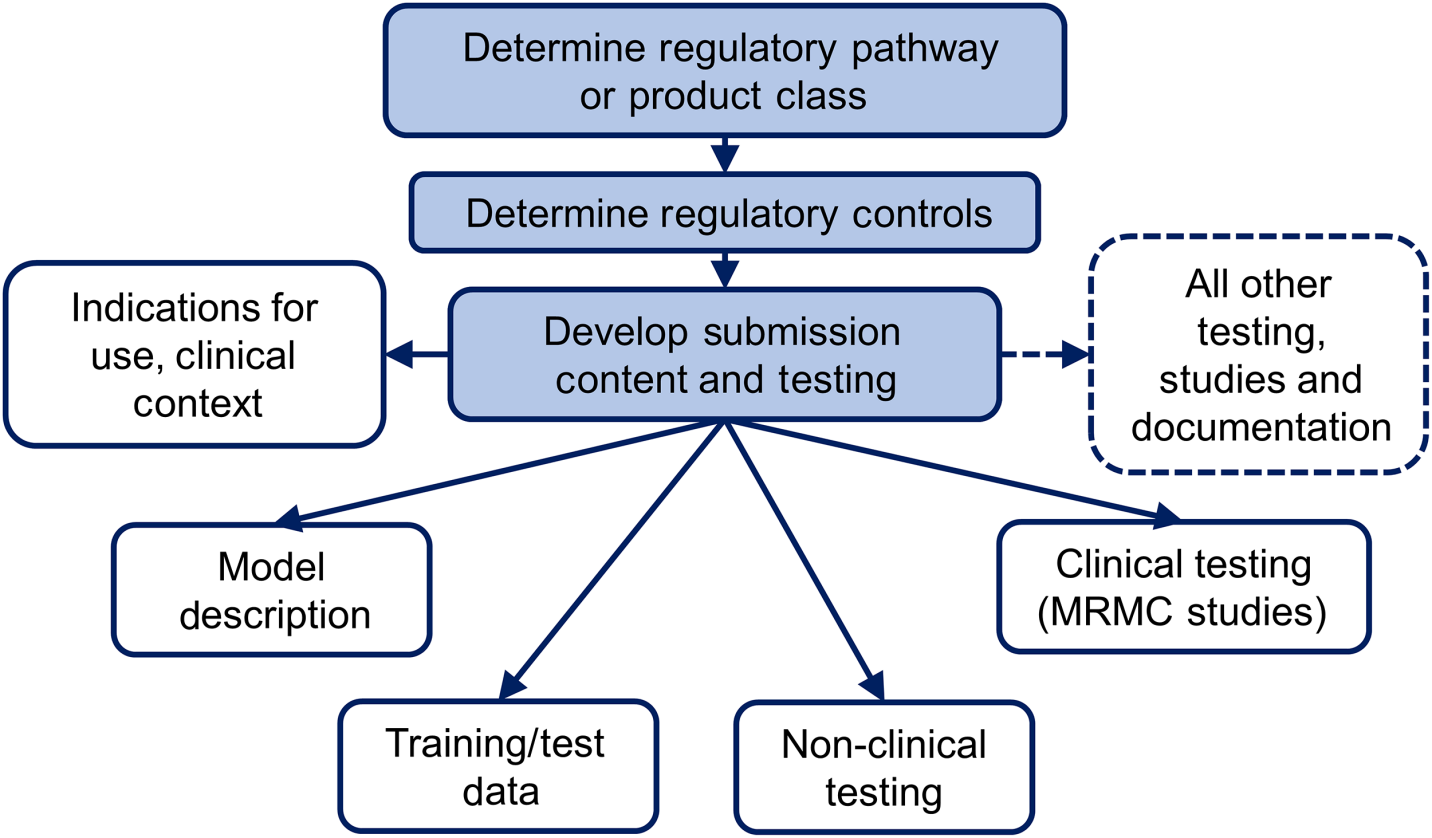
Flowchart depicting some common considerations for AI/ML device evaluation as part of a premarket submission.

Depending on the product class and technologies incorporated into the overall device, premarket AI/ML device submissions can contain a wide array of information and testing to facilitate the review process, e.g., a description of the device, a discussion of relevant standards conformed with, nonclinical studies documentation and testing, clinical studies documentation and testing, software documentation and testing, and cyber-security documentation and testing. Figure 1 shows some of the common considerations in AI/ML device assessment to be addressed as part of an AI/ML device submission. We will not discuss all the aspects of a premarket AI/ML device submission here, but we will discuss four critical aspects including model description, data, nonclinical testing, and multi-read multi-case testing.

### 2.2 Model Description

A conceptual description of an AI/ML model is a great start, but an engineering description of the architecture and how it was built is better. The description can include references to literature and figures with flow charts and diagrams. A detailed engineering description of the software helps reviewers understand the underlying functionality and complexity of the device, determine how it should be tested, and whether the performance of the device is expected to generalize to different data acquisition devices or patients. Details on the following are generally important to describe:

• input data and the dimension of each input, including patient images and patient meta data,
• engineered features and the feature selection processes, if appropriate,
• pre-processing and post-processing necessary for AI/ML model application,
• model network type(s) and components, e.g., model architecture including layers, activation functions, loss function, and the dimensionality of the data throughout the processing pipeline, and
• model development, including training and tuning processes, e.g., transfer learning, data augmentation, regularization methods, ensemble methods, tuning thresholds and hyper-parameters, optimization methods (optimality criteria), performance assessment metrics, calibration, and other documentable parameters.

### 2.3 Data

The data used in AI/ML training and testing are critical for developing robust models, especially when implementing a deep learning method that combines and automates feature extraction, feature selection, and classification. We refer to the patient images together with other patient and clinical information as data.^27^^,^^28^ Data for AI/ML device submissions come from a number of different sources including data collected by the manufacturer specifically for AI/ML device development and evaluation, other private data collection efforts, public data collections, and potentially even via synthetic data. Each of these collection sources has unique challenges and benefits related to burden, quality of the reference standard, representing the intended patient population, representing the intended image acquisition devices, and controlling access, e.g., preventing commingling of training and test data. The choice of data for any specific use or application is a tradeoff between these various factors. When collecting image data for developing or testing AI/ML models, it is important to also acquire appropriate clinical information, e.g., patient demographics, family history, reason for the exam; disease specific information, e.g., disease type and lesion size; image acquisition information, e.g., patient prep, device manufacturer and model, protocol, and reconstruction method; and other clinical test results in order to characterize and understand training and testing limitations and model generalizability. Providing tables and diagrams characterizing the data helps facilitate an efficient review process. Since developers often explore multiple model architectures and fine-tune parameters as part of the training process,^29^^,^^30^ it is useful to provide a flow chart of the entire development process and clarify the methods and data used in each step.

Dataset size is an important resource allocation issue. A general principle is for the training dataset to be large enough to allow an AI/ML model to learn the relationship between the input and output with little or no overfitting, and for the independent test dataset to be large enough to provide adequate precision of the performance estimates, i.e., sufficiently small error bars. The datasets, both training and test, should include relevant cohorts and subgroups containing enough patients/cases to facilitate robust algorithm training and facilitate subgroup analyses as discussed in Sec. 2.4. Research has shown that as the training set is gradually increased from a small size, overfitting initially decreases dramatically, with diminishing returns as the dataset size gets larger.^31^ The rate at which adding more data improves performance depends on the complexity of the AI/ML model and the complexity of the data space. Estimation of the test dataset size for adequate precision and study power is a classical problem in statistics, and pilot data are extremely helpful for estimating the dataset sizes appropriately.

#### 2.3.1 Independence

A central principle for performance evaluation is that the test dataset should be independent of the training dataset (different patients and different clinical sites) to avoid biases in performance assessment and demonstrate performance generalizability. Violation of this principle will result in optimistically biased performance estimates and potentially unacceptable real-world performance.^32^ Although this principle is simple, there are subtle ways in which the independence principle can be violated. A typical example of violation arises from the failure to recognize that multiple images (or image regions) from the same patient are correlated. The images from the same patient are different, but not independent. Therefore, to avoid information leakage between the training and testing datasets, the images from each patient should appear in only one of these datasets.

**Fig. 2:**
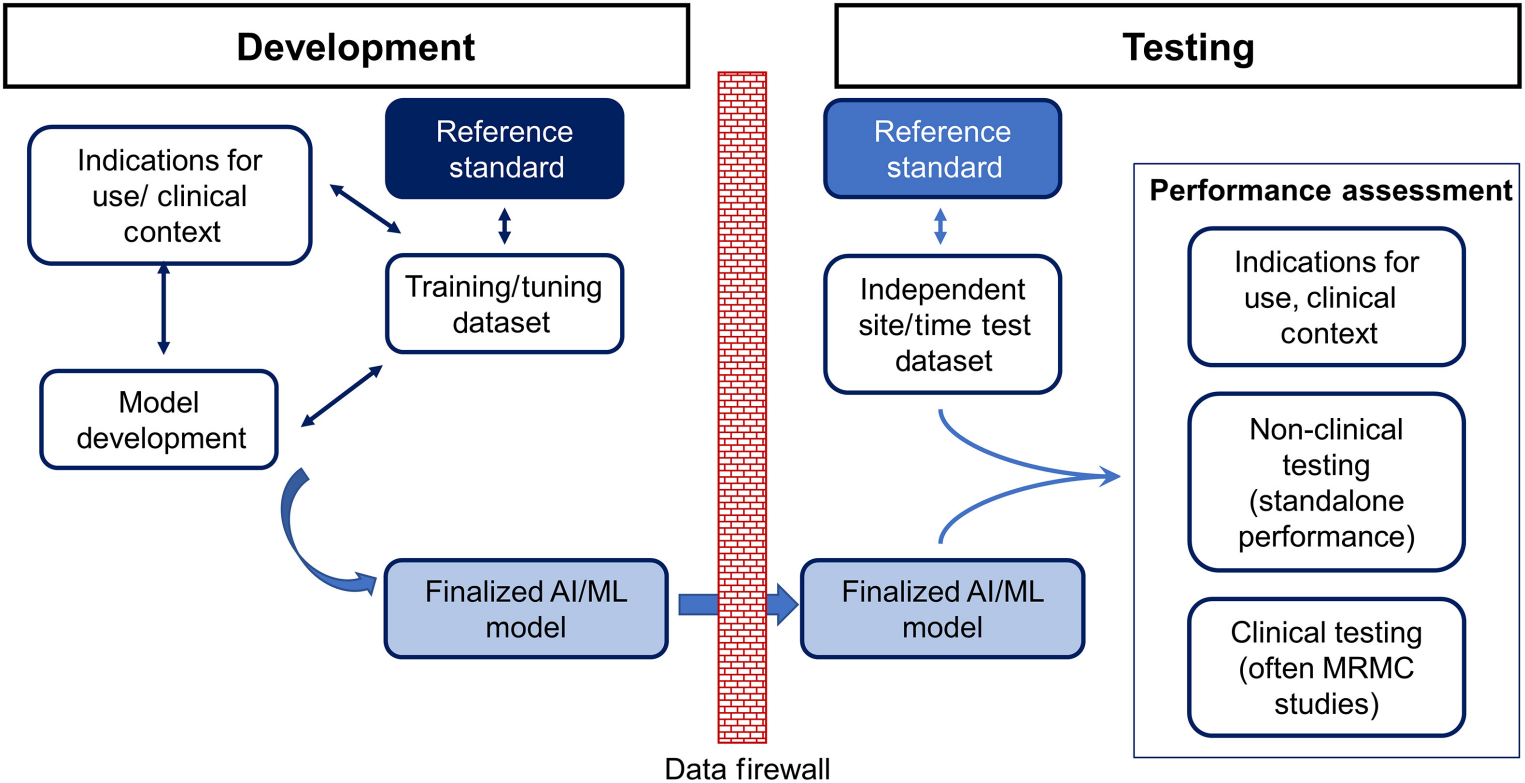
Flowchart depicting the AI/ML development process and its independence from the performance assessment conducted as part of a device submission. The test data are ideally site and time independent from that of the AI/ML training and tuning data.

A more subtle mechanism that can cause dependence between the training and test datasets arises when a single-site dataset is randomly split into training and testing datasets. This approach seems logical, but it may result in a site-specific similarity between the training and test datasets. Essentially, the training and testing data from the single site are more alike than what might be found in the real world such that this approach can underestimate overall variability, e.g., cross-site variability in data acquisition and clinical practice. When this approach is used, it is referred to as internal validation.^33^ The limitation of this approach can be mitigated by including data from multiple sites, but it is better to split the training and test data by site or at least by time. This approach is referred to as external validation.^34^ Figure 2 shows the need for the AI/ML development to be independent from the performance assessment conducted as part of a device submission including having the test data site and time independent from that of the AI/ML training and tuning data.

#### 2.3.2 Representativeness

The collected data should be large in size and representative of the target population. One approach is to collect consecutive cases that fit the intended use population from a diverse set of sites, e.g., academic, community, rural health providers, over a defined time period. This approach can be resource intensive when disease prevalence is low. Therefore, an alternative approach may be necessary, but it should still address the representativeness of the collected data as much as possible.

When collecting a training dataset, a key is to include a diverse set of cases. It may be efficient (improve model performance) to consider sampling methods that target “informativeness,” “representativeness,” or a combination of the two.^35^^,^^36^ For a testing dataset, it is more important to match the study data with the target population, but there may be some flexibility under FDA’s least burdensome principle^37^ in studies with controlled design and informed interpretation of results. It may be necessary to include rare cases or patient subgroups, or it may be statistically efficient to stratify, or enrich, the sampling across patient subgroups. If the enrichment is based solely on the disease condition, the impact may only be on the prevalence of the study set. Several performance metrics are unaffected by prevalence differences, e.g., sensitivity, specificity, and the area under the receiver operating characteristic (ROC) curve, denoted AUROC. Other differences between the study and clinical population can result in differences between reported device performance in the study and true performance on the clinical population, which may be problematic if not addressed. Questions about the appropriateness of testing datasets and assessment protocols are best addressed on a case-by-case basis in a Q-submission.

### 2.4 Nonclinical Testing, Standalone Performance

Nonclinical testing is a catchall category for performance testing that is not related to the active observation and treatment of patients.^38^ This includes mechanical, biological, and engineering performance (fatigue, wear, tensile strength) using *ex vivo*, *in vitro*, *in situ*, and simulation studies. For AI/ML devices, nonclinical testing may include software verification and validation, human factors validation, and standalone performance among other tests depending on the device and application area. Standalone performance is a measure of device performance alone with little to no interaction or interpretation from a clinical end user.^28^ When a clinical end user needs to interact with the device or interpret the device outputs, FDA generally requires an assessment of the device in the hands of the end users. Such an assessment happens in a “reader study,” which is discussed in Sec. 2.5.

Standalone testing also provides a performance benchmark for comparing AI/ML devices from the same or different manufacturers. This benchmark can reduce the need for clinical performance testing in future regulatory submissions. Putting aside the challenges related to establishing the reference standard, standalone testing is largely a computational exercise and can be systematically applied to a large number of sample cases. As such, standalone testing can be very useful for assessing device generalizability to different clinical subpopulations, sites, and to different image acquisition devices and protocols.^39^^,^^40^

#### 2.4.1 Evaluation metrics

Selection of performance metrics is crucial for benchmarking an AI/ML model and for comparing performance with predicate devices or other appropriate comparators. The endpoints selected to characterize standalone performance will depend on the clinical task (clinical endpoint) and type of AI/ML output being evaluated. However, selection of performance metrics is not trivial, and a single measurement may not completely benchmark performance or fully capture the model task. As discussed previously, some common type of medical imaging AI/ML devices include segmentation, CADx, CADe, and computer-aided triage (CADt). Sokolova et al. provides an overview of the different evaluation metrics by dividing AI/ML into binary, multi-class, multi-labeled, and hierarchical tasks.^41^ Other ways to categorize the metrics are based on whether the metric is prevalence dependent, applicable to binary truth, or applicable to multi-class truth.^42^ Which measurements may be most appropriate depends on the task for which the AI/ML model was developed, the scale associated with the truth and the AI/ML output,^43^ and which type of error should be most heavily weighted.^44^

#### 2.4.2 Subgroup analysis

Subgroup assessment is an important component of standalone testing. In this case, model performance is assessed on individual or combined subgroups to better understand where the model may have performance limitations. Studies may need to be sized to yield a certain statistical precision of the performance on different subgroups, or studies may simply report the performance on different subgroups with accompanying confidence intervals. For the most part, this information is used in a regulatory submission to label the device, but subgroup performance may be critical in establishing substantial equivalence or safety and effectiveness when specific performance claims are made for that subgroup. Depending on the clinical task and context, subgroup analyses found in AI/ML submissions are based on patient demographics, e.g., patient age, sex, race; image acquisition conditions, e.g., acquisition device and protocol; and disease type/presentation, e.g., disease subtype and lesion size/shape; or a combination of these characteristics.^31^^,^^45^

The subgroup analyses discussed above generally require prior knowledge of the important subgroups but some more automated techniques for identifying important subgroups, based on model audits^46^ and schema completion^47^ have been reported in the literature. Novel subgroup identification techniques may allow for generalizability analysis by automatically identifying important hidden stratifications impacting model performance.

#### 2.4.3 Repeatability and reproducibility

A repeatability or reproducibility study refers to standalone assessments that investigate differences in AI/ML output from reimaging a patient with the same or different acquisition devices and conditions. Such studies are commonly included in *in vitro* diagnostic device submissions and may have value for submissions of AI/ML devices.^48^^–^^50^ However, these designs have been less common in medical imaging applications, for example, when the study would have required exposing the patient to additional ionizing radiation. When appropriate, like scanning pathology slides multiple times with whole slide imaging systems or taking repeated pictures of a skin lesion with the camera of a mobile device, repeatability and reproducibility studies can demonstrate AI/ML device robustness and generalizability. More robust AI/ML devices will have higher repeatability/reproducibility across real-world use cases.

### 2.5 Multi-Reader Multi-Case Studies

AI/ML-enabled medical devices are often evaluated in the hands of clinicians especially when the intended use of the device is to assist clinicians in their clinical decision-making. Computer assistive AI/ML is particularly common in medical imaging applications. Here, we define a medical imaging reader study as a study in which readers, e.g., radiologists or pathologists, review and interpret medical images for a specified clinical task, e.g., diagnosis, and provide an objective interpretation, such as a rating of the likelihood that a condition is present. This is fundamentally different from a survey or questionnaire for the clinicians to indicate if they “like” some functionalities or features of an AI model, which is subjective and may not directly relate to how the AI/ML impacts clinician performance for a specific clinical task. When evaluating the clinical benefit of an AI/ML, it is ideal to have the conclusions of the clinical study generalize to both the intended patient population and the intended user population, i.e., readers. For this to occur, both readers and patient cases in the study should be representative of their respective populations, and both reader and case variability should be accounted for in the analysis method. The multi-reader multi-case (MRMC) study design is an approach that allows study conclusion to potentially generalize to both reader and patient populations when using the appropriate statistical analysis techniques.^51^^–^^53^

MRMC studies for medical imaging AI/ML typically consists of two arms: reading images without the AI/ML model and with the model for the clinical task for which the device is designed. This study allows reader performance with and without the AI/ML model to be compared. Many statistical methodologies for generalizing the performance to both the population of readers and the population of cases were developed including the Dorfman, Berbaum, and Metz (DBM) jackknife method,^54^^,^^55^ the Obuchowski and Rockkett (OR) ANOVA model-based method,^56^ the Beiden, Wagner, and Campbell bootstrap method,^57^ and the Gallas U-statistics method.^53^^,^^58^ While early developments of MRMC analysis methods focused on the area under the ROC curve (AUROC) as the preferred performance metric, most methods generalize to other endpoints, including binary outcome endpoints, e.g., the OR and U-statistics methods have been validated for binary outcome endpoints, such as sensitivity and specificity.^59^^,^^60^ Many of these MRMC statistical methods have publicly available software tools, such as the updated OR method^61^ and the U statistic method (iMRMC: software to do MRMC analysis of reader studies),^53^ making MRMC assessment possible for non-statisticians.

The design of an MRMC reader study involves a number of considerations including patient data collection, establishment of a reference standard, recruitment and training of readers, and the study design along with other factors. Recent work has demonstrated statistical and practical tradeoffs to be considered when assigning cases to readers (fully crossed versus split-plot designs).^62^^,^^63^ It is worth noting that MRMC studies for the assessment of imaging-based AI/ML are often retrospective and controlled “laboratory” studies, in which only the images and information related to the device of interest is presented to the readers, e.g., “image only” versus “image plus AI/ML output.” This study approach is not fully consistent with the clinical reading scenario as physicians often have more information available, e.g., patient history, other clinical tests, and/or imaging exams. The studies are also often enriched with diseased cases when the natural prevalence of disease is low. The purpose of these design choices is to focus more directly on the AI/ML aid by limiting the impact of certain confounders and increasing the statistical power of the study rather than directly studying the “absolute” performance of clinicians in the real world.

While these specific design choices are often, but not always, acceptable in assessing the clinical performance of an AI/ML aid, efforts should be made to ensure the execution of the MRMC study is as close as possible to the clinical environment and identify/mitigate potential biases. For example, readers should be trained to appropriately use the AI/ML device, as this will help ensure the study design is not impacted as much by the readers learning on the fly how to use the AI/ML information effectively. In some cases, a prospective MRMC study may be necessary to clinically test an AI/ML device. For example, colonoscopy AI/ML aids have been assessed using a two-armed prospective MRMC study designs that more directly assesses the clinical impact of the AI/ML aid in device submissions.^64^^,^^65^ It is also important to randomize cases, readers, and reading sessions to minimize bias. For more details on the design of MRMC studies, interested readers can refer to an FDA guidance document,^66^ a consensus paper by Gallas et al.,^51^ as well as a tutorial paper by Wagner et al.^52^

## 3 Discussion

There are many challenges to producing high quality medical imaging AI/ML that can effectively translate into clinical use. Saw et al. identified four overarching challenges for effectively implementing medical imaging AI/ML: data governance, algorithm robustness, stakeholder consensus, and legal liability.^67^ While many of these challenges are intimately part of an AI/ML device review (data governance, algorithm robustness, and performance assessment), others, such as legal liability, generally fall outside of FDA’s purview. Data governance concerns relate to developing effective policies and protocols for storing, securing, and maintaining data quality, including images, metadata, and reference standard (truth) labels.^67^ Algorithm robustness concerns generally include how to reduce algorithmic bias and improve fairness across patients, groups, and sites. FDA is particularly concerned with how sponsor studies, often based on limited patient, group and site diversity, generalize to actual clinical practice across the United States. All AI/ML algorithms are biased to some extent because the data used to train the models are intrinsically a function of the population groups, disease conditions, clinical environments, and imaging technologies available during the collection process.^67^ Therefore, robustness includes not only developing methods to measure and reduce important sources of bias but also defining fairness criteria for the AI/ML under appropriate operational conditions as well as integrating the appropriate level of transparency. Methodological assessment is also a critical aspect of the FDA review process. There are a large number of potential performance metrics, statistical approaches, and baseline comparators available for assessing AI/ML devices.^68^ The challenge is to then determine the most meaningful data, metrics, methodological approaches, and success criteria for each unique AI/ML application while also controlling information leakage from the performance evaluation back into the device development process.

Many of these challenges are daunting and require contributions from developers, researchers, clinicians, patients, and regulatory communities. The agency’s regulatory thinking and processes are evolving to address at least some of these challenges. One area of current interest is when should an AI/ML SaMD require a premarket submission for an algorithm change, including how to best regulate continuously learning AI/ML devices. FDA released a discussion paper on this topic in 2019.^69^ This discussion paper proposed a framework for regulating modifications to AI/ML-based SaMD that relies on the principle of a “predetermined change control plan” (PCCP). The discussion paper was a way to seek early input from groups and individuals outside the agency on this topic prior to development of any draft guidance document. FDA then released an AI/ML-based software as a medical device action plan in early 2021.^70^ The action plan supports FDA’s commitment to developing innovative approaches for regulating medical device software and other digital health technologies and was developed in direct response to the feedback received on the discussion paper. The FDA identified multiple challenges around regulating AI/ML and five main actions to pursue. These were^70^

(1) updating the proposed framework for AI/ML-based SaMD, including issuance of a draft guidance document on PCCPs,
(2) encouraging development and harmonization of good machine learning practices (GMLPs),
(3) holding a public workshop on medical device labeling to support transparency for users of AI/ML devices,
(4) supporting regulatory science efforts on the evaluation and improvement of AI/ML, and,
(5) advancing real-world performance pilots to provide additional clarity on what a real-world evidence generation program for AI/ML could look like.

FDA provides the device development community with guidance documents that represent FDA’s current thinking and policies on regulatory issues (21 CFR 10.115(b)).^71^ Guidance documents most often not only relate to the design, production, labeling, promotion, manufacturing, and testing of regulated products but can also discuss the processing, content, and evaluation, approval of submissions, or inspection and enforcement policies. The agency has two seminal guidance documents related to radiological imaging-based AI/ML devices. These documents discuss premarket notification [510(k)] submission details^72^ and clinical performance assessment^66^ of computer-assisted detection devices applied to radiology images and device data. FDA has a number of other potentially relevant guidance documents including guidance documents describing recommendations for performance data and software documentation for SaMD devices,^73^ software validation,^74^ and technical performance assessment for quantitative imaging devices.^75^ In response to the 2021 AI/ML-based software as a medical device action plan, the FDA developed and recently released a draft guidance document entitled “Marketing Submission Recommendations for a Predetermined Change Control Plan for Artificial Intelligence/Machine Learning (AI/ML)-Enabled Device Software Functions.”^13^ This draft guidance document provides a framework for addressing some types of AI/ML algorithm modifications through a PCCP and is now available for review. FDA is encouraging individuals and organizations to submit comments for the FDA to consider before this guidance document is finalized.

To address the challenge around defining core GMLP concepts, FDA, Health Canada, and the United Kingdom’s Medicines and Healthcare products Regulatory Agency identified 10 guiding principles for the development of GMLP that help promote safe, effective, and high-quality AI/ML.^12^ The agency is also participating in collaborative communities,^76^ such as the AFDO/RAPS healthcare products collaborative community, which is focused on GMLP development, and the pathology innovation collaborative community, where AI/ML is a major topic driving community activities.

Many groups have recognized a lack of transparency in AI/ML-enabled medical devices. For example, van Leeuwen et al.^77^ determined that only 36/100 identified European CE-marked AI/ML products had peer-reviewed performance information available with only 18/36 demonstrating at least some potential clinical impact. FDA makes device summaries available for all cleared/approved medical devices, including medical imaging AI/ML, but these summaries may lack some types of information needed by specific stakeholders. Transparency is not only a concern for regulators but across the AI/ML landscape. One example of this is a lack of code and data sharing hindering the ability of outside groups to perform reproducibility studies and assessments for many AI/ML algorithms appearing in the peer-reviewed literature.^78^ Therefore, there remains transparency challenges including determining exactly what information is needed, who needs it and when, and how best to present this information to the audience. The FDA held a virtual public workshop on the transparency of AI/ML-enabled medical devices in October, 2021, to hear from stakeholders on considerations for achieving transparency for users of AI/ML-enabled medical devices, and to gather input on the types of information that would be helpful for a manufacturer to include in device labeling and public facing documents to support transparency and potentially AI/ML explainability.^79^

Many AI/ML performance assessment and algorithm robustness challenges still depend on the development of novel approaches and tools. The Office of Science and Engineering Labs (OSEL) within CDRH is focused on advancing AI/ML regulatory science.^14^ Regulatory science is the science of developing new tools, standards, and approaches to assess the safety, efficacy, quality, and performance of FDA-regulated products. OSEL has an AL/ML regulatory science program conducting research to ensure patients have access to safe and effective medical AI/ML.^80^ This research is addressing major scientific gaps and challenges including (a) a lack of methods for the enhancement of AI/ML model training for clinical datasets that are typically much smaller than nonclinical datasets, (b) a lack of clear definitions or understanding of artifacts, limitations, and failure modes for deep-learning models in the denoising and reconstruction of medical images, (c) a lack of a clear reference standard for assessing the accuracy of AI/ML-based quantitative imaging and radiomics tools, (d) lack of assessment techniques to evaluate the trustworthiness of adaptive and autonomous AI/ML devices, e.g., continuously learning models, and (e) a lack of systematic approaches to address the robustness of various AI/ML input factors, such as data acquisition factors, patient demographics, and disease factors, to patient outcomes in a regulatory submission.

The AI/ML research program^80^ strives to address these challenges by conducting peer-reviewed research to develop and understand methods for enhanced AI/ML training, developing systematic approaches for understanding AI/ML robustness, and assessing novel test methodologies to evaluate fixed and continuously learning AI/ML performance in both the premarket and real-world settings, to name just a few areas of ongoing research.

Some of the regulatory science projects being conducted as part of the OSEL AI/ML research program include a recent investigation developing a cooperative labeling technique to incorporate weakly labeled data into the training of a deep learning AI/ML model for lung nodule detection in CT.^81^ This study showed that the inclusion of weakly labeled data leads to a 5% improvement in lung nodule detection performance when the number of expert annotations is limited. Another approach for addressing small dataset sizes is to augment available data with synthetic datasets. Cha et al.^82^ compared detection performance when the network was trained using different percentages of real and synthetic mammograms. Synthetic mammograms were generated using *in silico* breast and lesion models followed by the creation of synthetic mammograms of the breast models. The results showed that a statistically significant improvement in detection sensitivity can be achieved when synthetic images are added to real mammograms in algorithm training. These results indicate that novel approaches for augmenting and expanding training data, e.g., using generative, *in silico* or phantom-based augmentation, could play a role in reducing burden and improving the performance and robustness of medical imaging AI/ML devices.

OSEL is also exploring approaches for efficiently and effectively utilizing limited data in AI/ML algorithm modifications. A Centers of Excellence in Regulatory Science and Innovation (CERSI) collaboration between the University of California at San Francisco and OSEL investigated whether an online logistic recalibration and revision procedure can be designed with performance guarantees on updates to an original “static” AI/ML model. The overall goal was to avoid the risk of deteriorating model performance that may inadvertently result from an AI/ML model update. The team designed two procedures for continual recalibration or revision of an underlying AI/ML model. These procedures guarantee that the updated models are noninferior to the static model and often produce model revisions that improve over time as is desired.^83^ Result from an empirical evaluation via simulations and a real-world study predicting chronic obstructive pulmonary disease risk showed that both methods outperformed the static model and other common online revision techniques.

Another OSEL regulatory science effort is examining the expected time saving from a CADt device implemented within a clinical setting. Ideally, a CADt device prioritizes patients with a time sensitive condition so these patients are evaluated more quickly. However, quantifying the time savings is challenging because of the complex and heterogeneous clinical environments a CADt device may be used in. OSEL scientists have developed a theoretical method, based on queueing theory, to quantify the wait-time-savings of CADt in various clinical settings.^84^ The theoretical model was validated via simulation studies and allows model users to investigate CADt performance under various clinical settings, including changes in disease prevalence, patient arrival rate, radiologist reading rate, number of radiologists on-site, and the presence of emergency patient images.

OSEL is also developing statistical methods for assessing AI/ML device performance. For example, an agreement endpoint may be the most acceptable metric for assessment of AI/ML devices that output a quantitative measurement derived from a medical image, especially when the reference method is clinicians estimating the same value. A recent OSEL paper reports on a three-way mixed effects ANOVA technique for estimating MRMC agreement in a statistically rigorous manner.^85^ Another recent study investigated a method for controlling “information leakage” through, for example, the repeated reuse of test data in AI/ML device evaluation studies. Test data reuse is related to the problem of privacy loss due to repeated queries of information from a database. Differential privacy methods have been developed to address the latter problem and have also been applied to the former test data reuse problem as well. This OSEL study^86^ extended the reusable holdout mechanism of Dwork et al.^87^ to the more common AUROC endpoint used in AI/ML device assessment and showed that this method substantially reduced overfitting to the test data, even when the test dataset is small, but comes at the cost of increased uncertainty in the reported performance.

To accelerate the transfer of regulatory science methods into the fast-evolving AI/ML technological landscape, outcomes from these research efforts are being released as regulatory science tools (RSTs).^88^ RSTs are peer-reviewed computational or physical phantoms, methods, datasets, computational models, and simulation pipelines designed to support the assessment of safety or effectiveness of a medical device or emerging technology. These tools are well characterized for their applications and are made broadly available through a CDRH/OSEL public catalog of more than 100 RSTs, including AI/ML assessment tools.^88^ One available AI/ML assessment RST is the iMRMC tool that can be used to assist investigators in analyzing and sizing MRMC reader studies.^53^ This catalog is being expanded as new tools are developed.

## 4 Conclusion

This paper discussed FDA medical device review processes, including device types, product classifications, and regulatory pathways for medical imaging AI/ML devices. The device class (classes I, II, or III) and the regulatory pathway (PMA, 510k, or De Novo) are based on the level of risk associated with an AI/ML device and informed by both the technological characteristics and intended use of the device. Over five hundred medical devices incorporating AI/ML technology have been granted marketing authorization by the FDA through a combination of the PMA, 510k, and De Novo regulatory pathways, with the majority of these devices analyzing radiological image data. Even though the history of AI/ML devices on the market is long, AI/ML is still a fast-changing and evolving technology that presents novel regulatory challenges for developing robust assessment methods and providing effective regulatory oversight. To solve these challenges, FDA is conducting and facilitating AI/ML regulatory science research that focuses on allowing innovation to flourish through least burdensome regulatory methods while still assuring that patients have timely and continued access to safe, effective, and high-quality AI/ML devices.

## Disclaimers

The mention of commercial products, their sources, or their use in connection with material reported herein is not to be construed as either an actual or implied endorsement of such products by the Department of Health and Human Services. This is a contribution of the U.S. Food and Drug Administration and is not subject to copyright.
